# Supplementary material for: Modeling glioblastoma heterogeneity as a dynamic network of cell states
Source: Mol Syst Biol. 2021 Sep 16;17(9):e10105. doi: 10.15252/msb.202010105 (PMC8444284; doi:10.15252/msb.202010105)
Supplement: Supplementary file 6 — Source Data for Figure 5 [file MSB-17-e10105-s004.zip › Figure5A_sourcedata/GSEA_3017/nosigdb_stateC.GseaPreranked.1622795002785/SUVA_MES1_PMID_31327527.html]

Details for gene set SUVA\_MES1\_PMID\_31327527[GSEA]

|  || Dataset | state13017 |
| Phenotype | NoPhenotypeAvailable |
| Upregulated in class | na\_neg |
| GeneSet | SUVA\_MES1\_PMID\_31327527 |
| Enrichment Score (ES) | -0.6603537 |
| Normalized Enrichment Score (NES) | -2.8375645 |
| Nominal p-value | 0.0 |
| FDR q-value | 0.0 |
| FWER p-Value | 0.0 |
Table: GSEA Results Summary

  

Fig 1: Enrichment plot: SUVA\_MES1\_PMID\_31327527      
 Profile of the Running ES Score & Positions of GeneSet Members on the Rank Ordered List

  

| PROBE | GENE SYMBOL | GENE\_TITLE | RANK IN GENE LIST | RANK METRIC SCORE | RUNNING ES | CORE ENRICHMENT || 1 | IGFBP7 |  |  | 225 | -0.259 | -0.3633 | No |
| 2 | CLIC1 |  |  | 237 | -0.261 | -0.3555 | No |
| 3 | NAMPT |  |  | 306 | -0.280 | -0.4446 | No |
| 4 | SERPINE1 |  |  | 323 | -0.286 | -0.4430 | No |
| 5 | S100A11 |  |  | 361 | -0.301 | -0.4761 | No |
| 6 | TIMP1 |  |  | 399 | -0.320 | -0.5073 | No |
| 7 | WWTR1 |  |  | 437 | -0.345 | -0.5359 | No |
| 8 | LGALS3 |  |  | 471 | -0.374 | -0.5546 | No |
| 9 | IFITM3 |  |  | 533 | -0.477 | -0.6113 | Yes |
| 10 | RCAN1 |  |  | 544 | -0.505 | -0.5768 | Yes |
| 11 | GJA1 |  |  | 552 | -0.527 | -0.5347 | Yes |
| 12 | ANXA2 |  |  | 563 | -0.567 | -0.4937 | Yes |
| 13 | MT2A |  |  | 566 | -0.575 | -0.4380 | Yes |
| 14 | EMP3 |  |  | 568 | -0.580 | -0.3801 | Yes |
| 15 | S100A16 |  |  | 569 | -0.582 | -0.3202 | Yes |
| 16 | CD44 |  |  | 575 | -0.659 | -0.2611 | Yes |
| 17 | SPP1 |  |  | 577 | -0.674 | -0.1935 | Yes |
| 18 | VIM |  |  | 578 | -0.674 | -0.1241 | Yes |
| 19 | S100A10 |  |  | 582 | -0.691 | -0.0582 | Yes |
| 20 | EMP1 |  |  | 589 | -0.785 | 0.0121 | Yes |
Table: GSEA details [plain text format]

  

Fig 2: SUVA\_MES1\_PMID\_31327527: Random ES distribution      
 Gene set null distribution of ES for **SUVA\_MES1\_PMID\_31327527**
